# Canonical and noncanonical Wnt pathways: a comparison between endometrial cancer type I and atrophic endometrium in Brazil

**DOI:** 10.1590/S1516-31802011000500007

**Published:** 2011-09-01

**Authors:** Marina de Pádua Nogueira Menezes, Celina Tizuko Fujiyama Oshima, Levon Badiglian, Thiago Simão Gomes, Luis Fernando Mesias Barrezueta, João Norberto Stávale, Wagner José Gonçalves

**Affiliations:** I MD. Gynecologist and Gynecological and Obstetric Surgeon, Gynecological Oncology Sector, Department of Gynecology, Universidade Federal de São Paulo — Escola Paulista de Medicina (Unifesp-EPM), São Paulo, Brazil.; II MD, PhD. Adjunct Professor, Department of Pathology, Universidade Federal de São Paulo — Escola Paulista de Medicina (Unifesp-EPM), São Paulo, Brazil.; III MSc. Biologist, Department of Pathology, Universidade Federal de São Paulo — Escola Paulista de Medicina (Unifesp-EPM), São Paulo, Brazil.; IV MSc. Pathologist, Department of Pathology, Universidade Federal de São Paulo — Escola Paulista de Medicina (Unifesp-EPM), São Paulo, Brazil.; V MD, PhD. Adjunct Professor, Head of the Gynecological Oncology Sector, Department of Gynecology, Universidade Federal de São Paulo — Escola Paulista de Medicina (Unifesp-EPM), São Paulo, Brazil.

**Keywords:** Wnt proteins, Endometrial neoplasms, Women, Postmenopause, Endometrium, Proteínas Wnt, Neoplasias do endométrio, Mulheres, Pós-menopausa, Endométrio

## Abstract

**CONTEXT AND OBJECTIVE:**

The Wnt pathway is involved in tumorigenesis of several tissues. For this reason, we proposed to evaluate Wnt gene expression in endometrial cancer type I.

**DESIGN AND SETTING:**

Cross-sectional study on materials gathered from the tissue bank of the Department of Pathology, Universidade Federal de São Paulo.

**METHODS:**

Endometrial specimens were obtained from surgeries performed between 1995 and 2005 at São Paulo Hospital, Universidade Federal de São Paulo. The material was divided into two groups according to tissue type: Group A, atrophic endometrium (n = 15); and Group B, endometrial adenocarcinoma (n = 45). We compared the immunohistochemical expression of Wnt1, Frizzled-1 (FZD1), Wnt5a, Frizzled-5 (FZD5) and beta-catenin between endometrial cancer type I and atrophic endometrium.

**RESULTS:**

Regarding Wnt1, FZD1 and Wnt5a expression, no significant association was observed between the groups. A significant association was observed between the groups in relation to FZD5 expression (P = 0.001). The proportion of FZD5-positive samples was significantly higher in group A (80.0%) than in group B (31.1%). Regarding the survival curve for FZD5 in group B, we did not find any significant association between atrophic endometrium and endometrial adenocarcinoma. We also did not find any significant association regarding beta-catenin expression (P = 1.000).

**CONCLUSION:**

FZD5 is downregulated in endometrial adenocarcinoma, in comparison with atrophic endometrium.

## RESUMO

**CONTEXTO E OBJETIVO:**

A via Wnt está envolvida na tumorigênese de diversos tipos de tecidos. Por essa razão, propusemo-nos a avaliar a expressão de genes da família Wnt no câncer endometrial tipo I.

**TIPO DE ESTUDO E LOCAL:**

Estudo transversal com coleta de materiais do banco de tecidos do Departamento de Patologia da Universidade Federal de São Paulo.

**MÉTODOS:**

Amostras endometriais foram obtidas de cirurgias que ocorreram entre 1995 e 2005 no Hospital São Paulo, Universidade Federal de São Paulo. Foram separados dois grupos segundo o tipo de tecido obtido: grupo A, com endométrio atrófico (n = 15); e grupo B, com adenocarcinoma endometrial (n = 45). Comparamos a expressão imunoistoquímica de Wnt 1, Frizzled-1 (FZD1), Wnt 5a, Frizzled-5 (FZD 5) e beta-catenina entre câncer endometrial tipo I e endométrio atrófico.

**RESULTADOS:**

Na expressão do Wnt1, FZD1 e Wnt5a, não observamos associação significante entre os grupos. Na expressão do FZD5, encontramos associação significante entre os grupos (P = 0,001). A proporção de positividade do FZD5 foi significantemente maior no grupo A comparado ao grupo B (31,1%). Em relação à curva de sobrevida para o FZD5 no grupo B, não tivemos associação significante entre endométrio atrófico e adenocarcinoma do endométrio. Também não observamos associação significante na expressão da beta-catenina (P = 1,000).

**CONCLUSÃO:**

FZD5 é *downregulated* no adenocarcinoma endometrial quando comparado ao endométrio atrófico.

## INTRODUCTION

The Wnt family has an important role in tumorigenesis and embryogenesis.^1-5^ Wnts are an evolutionarily highly conserved family of genes/proteins that act through three signaling pathways.^6^ The canonical pathway involves regulation of beta-catenin. Briefly, in the absence of Wnt signaling, a multiprotein complex that includes adenomatous polyposis coli (APC), glycogen synthase kinase-3 (GSK3) and axin ensures degradation of beta-catenin, thereby limiting the free intracytoplasmic pool of beta-catenin. The presence of Wnt signaling through the Frizzled (FZD) receptor and the low-density lipoprotein receptor-related protein 5 and 6 (LRP5/6) receptor complex inactivates GSK3 and causes its dissociation from axin, thereby preventing phosphorylation of beta-catenin. The intracytoplasmic pool of beta-catenin thus increases, and it translocates to the nucleus, where it complexes with members of the T-cell factor/lymphocyte enhancement factor (LEF/TCF) family of transcription factors to mediate transcriptional induction of target genes such as c-myc, cyclin D, vascular endothelial growth factor (VEGF) and others.^1-5^

In noncanonical or planar cell polarity (PCP) signaling, Wnt signaling is transduced through FZD, independent of LPR5/6. This pathway mediates cytoskeletal changes through activation of the small GTPases Rho and Rac. Another noncanonical Wnt signaling pathway is WntCa2+. Wnt signaling via FZD mediates activation of heterotrimeric G-proteins, which engage Dsh, phospholipase C calcium-calmodulin kinase 2 (CamK2) and protein kinase C (PKC). This pathway modulates cell adhesion and motility.^7^

A fourth Wnt signaling pathway can be envisaged, since Chen et al. demonstrated that adenylyl cyclase signaling via protein kinase A (PKA) and its target transcription factor cAMP-responsive element-binding protein (CREB) are required for Wnt-directed myogenic gene expression. They also showed that Wnt proteins can also stimulate CREB-mediated transcription.^6,8^

In relation to endometrial cancer, most of the studies that have linked Wnt signaling to this disease focused on the role of beta-catenin. However, other components of Wnt signaling have been highlighted in recent published papers.

## OBJECTIVE

To investigate the role of the expression of the proteins Frizzled-1, Wnt5a, Frizzled-5 and beta-catenin on atrophic endometrial tissues and endometrial cancer, using immunohistochemical techniques on tissue microarrays obtained from postmenopause women.

## METHODS

Endometrial specimens were obtained from operations performed between 1995 and 2005 in the Gynecological Oncology Sector of the Universidade Federal de Sao Paulo (Unifesp), from patients who underwent laparotomy to treat endometrial cancer or hysterectomy to treat benign disease. All tissue samples obtained over that period were included in the study (convenience sample limited by time). None of the patients had received any preoperative therapy. We did not have any sample losses because this was a cross-sectional study.

The patients were divided into two groups: Group A, atrophic endometrium (n = 15); and Group B, endometrial adenocarcinoma (n = 45).

All the patients had menopausal status at the time of diagnosis. All the patients in group B had the endometrioid histological subtype of endometrial adenocarcinoma, at a variety of tumor grades. The cases of atrophic endometrium (group A) were compared with the endometrial cancer cases Group B, using parameters that avoided hormonal interference during the tissue analysis, such as exclusion of menopausal women.

The clinical and surgical staging and histological typing were performed in accordance with the classification of the International Federation of Gynecology and Obstetrics.^9^ The study was approved by the Institutional Ethics Committee (Unifesp).

## RESULTS

**Figure 1.**
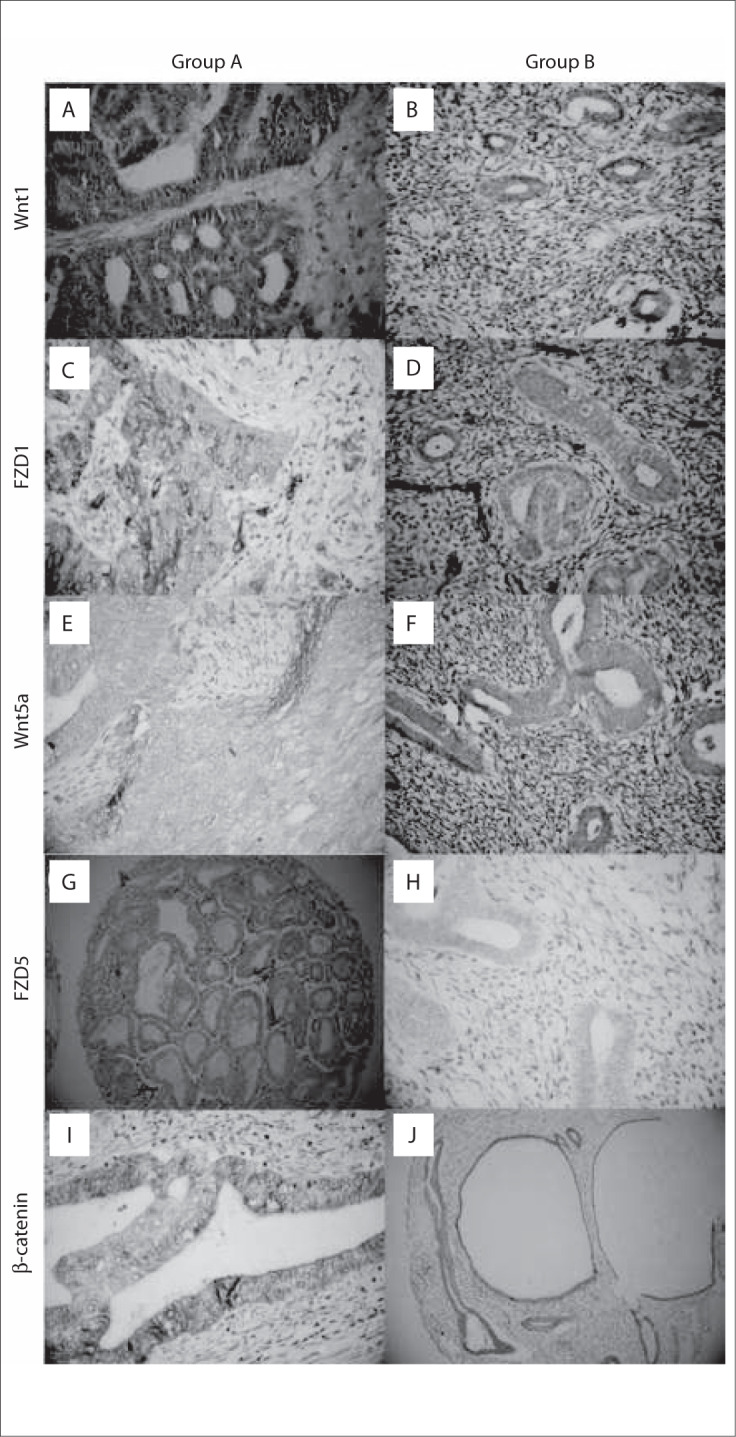
Expression of the proteins Wnt1, Wnt5a, Frizzled-1 (FDZ1), Frizzled-5 (FDZ5) and beta-catenin in groups A and B.

Most of the patients in group B were staged as IB and IC (Table 1). In all cases, expression of the markers was found practically only in the cytoplasm (Figure 1).

**Table 1.** Clinical staging of patients with endometrial cancer

Clinical staging – n (%)	n = 44
IB	13 (29.5%)
IC	16 (36.4%)
IIA	2 (4.5%)
IIB	5 (11.4%)
IIIA	5 (11.4%)
IIIB	1 (2.3%)
IIIC	2 (4.5%)

Regarding Wnt1, FZD1 and Wnt5a expression, no significant difference was observed between the groups.

**Figure 2.**
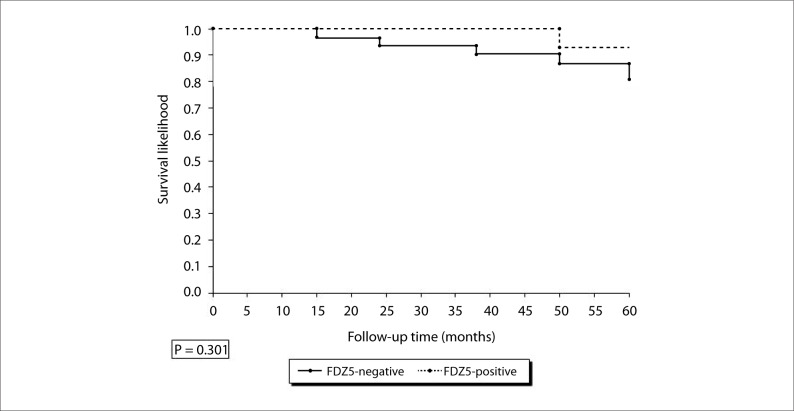
Survival curve for Frizzled-5 (FDZ5) in groups A and B.

A significant difference was observed between the groups in relation to FZD5 expression (P = 0.001). The proportion of FZD5-positive women was significantly higher in group A (80.0%) than in group B (31.1%). Regarding the survival curve for FZD5 in group B, we did not find any significant difference between positive and negative women (Figure 2).

No significant difference in beta-catenin expression was observed between patient groups, since the expressions for groups A and B were 100% and 95.6%, respectively (P = 1.000).

## DISCUSSION

### Canonical pathway

Endometrioid endometrial adenocarcinoma is essentially connected with type I endometrial cancer, which in turn correlates with hyperestrogenism status. Hence, the key to understanding the role of Wnt signaling in type I endometrial cancer is to find the link between Wnts and estrogen signaling. Many authors have suggested that a mutation of beta-catenin/*CTNNB1* in the endometrium would lead to nuclear accumulation of beta-catenin and then result in uterine endometrioid cancer.^1-3^

Pijnenborg et al.^3^ found nuclear accumulation of beta-catenin in 38% of their endometrioid endometrial cancer cases, while Kariola et al. found 53% and Schlosshauer et al. found 47%.^4,5^ On the other hand, we found no difference in beta-catenin expression between cases of atrophic endometrium and endometrial cancer, since both groups stained almost equally in the cytoplasm. This may be explained by the fact that beta-catenin has a low level of association with endometrial cancer, compared with loss of PTEN (phosphatase and tensin homologue protein).^6^ Furthermore, it seems that PTEN mutations do not cause nuclear beta-catenin accumulation in endometrial carcinomas.^7^

It is possible that beta-catenin has a more important role in relation to precursor lesions than in uterine cancer itself. Hideyuki et al. found nuclear beta-catenin in 70% of their endometrial hyperplasia samples and 56.7% of their endometrial cancer samples. However, they also showed that nuclear staining of beta-catenin occurred in the mid-proliferative, late proliferative and early secretion phases of the normal endometrium during the menstrual cycle. Twelve out of 15 cases (80.0%) during these periods showed nuclear staining.^8^

All these findings are concordant with reports that correlate estrogens with the canonical Wnt pathway, thus suggesting that estrogens or estrogen receptors are capable of stimulating beta-catenin accumulation.^10,11^

Comparing lean and obese rats, Zhang et al. found that estrogen more strongly induced the antiproliferative genes retinaldehyde dehydrogenase-2 and secreted frizzled-related protein-4 (a negative regulator of Wnt signaling) in lean rats, but had little or no effect on obese rats.^12^

### Noncanonical pathway

Both groups reached total staining for Wnt5a, and the proportion of FZD5-positive women was significantly higher in group A (80.0%) than in group B (31.1%).

It is known that noncanonical signaling can inhibit canonical signaling through a variety of mechanisms.^13^ We suggest that because of the low proportion of FZD5 positivity in the adenocarcinoma group, the noncanonical signaling could not function properly and that this subsequently led to development of hyperplasia and cancer.

Singleton et al. found that Wnt5a and FZD5 were downregulated by bisphenol-A (an estrogen receptor agonist) and estradiol.^14,15^ These findings are concordant with the physiopathology of type I endometrial adenocarcinoma.

Breast cancer is comparable with endometrial cancer in that it is also stimulated by estrogens. Jönsson et al. found that loss of Wnt5a protein expression was significantly associated with higher histological grade, recurrent disease, early relapse and death.^16^ Similar findings have been reported by others.^16-18^

Recently, we advocated the idea that the noncanonical Wnt pathway has an important role in ovarian cancer and would lead to a worse prognosis. Furthermore, we advocated that beta-catenin would not play an important role in epithelial ovarian cancer.^19^ However, endometrioid ovarian cancer is an exception to this. This neoplasia has a molecular pattern similar to that of endometrial adenocarcinoma, with regard to the Wnt pathway. In fact, in this respect, we suggest that low activity of the noncanonical Wnt pathway would lead to type I endometrial adenocarcinoma. This would reflect the complexity of the Wnt pathway, in which the concepts of tumor suppressors and promoters are fickle. Although we did not find any difference between the groups regarding Wnt5a, others have found that it was downregulated in endometrial carcinomas, in comparison with normal tissue.^20^

Regarding the survival curve for FZD5, there was a tendency towards better prognosis for FZD5-positive women, but this association was not significant. This also strengthens the idea that the noncanonical Wnt pathway has a protective function in relation to endometrial carcinoma. The more we learn about the Wnt family and its interactions, the more we obtain new insights into old problems.

## CONCLUSION

Wnts can produce different effects depending on the context. Here, we showed that FZD5 is downregulated in type I endometrial adenocarcinoma, compared with atrophic endometrium.
